# Non-Entomopathogenic Roles of Entomopathogenic Fungi in Promoting Plant Health and Growth

**DOI:** 10.3390/insects10090277

**Published:** 2019-09-01

**Authors:** Surendra K. Dara

**Affiliations:** Division of Agriculture and Natural Resources, University of California Cooperative Extension, San Luis Obispo, CA 93401, USA; skdara@ucdavis.edu; Tel.: +1-805-720-1700

**Keywords:** *Beauveria*, *Isaria*, *Metarhizium*, entomopathogenic fungi, endophyte, mycorrhiza-like, systemic resistance

## Abstract

Multiple genera of hypocrealean fungi infect and kill a wide variety of arthropod pests. Several formulations based on these soilborne fungi are commercially available as biopesticides for controlling urban, garden, greenhouse, and agricultural pests. These fungi are an important part of integrated pest management strategies to maintain pest control efficacy, reduce the risk of chemical insecticide resistance, and offer environmentally sustainable pest suppression. While the entomopathogenic or pest management role of these fungi is well documented, several studies in the past decade or two have provided insights into their relationship with plants, soil, and plant pathogens, and their additional roles in promoting plant growth and health. This review highlights these endophytic, mycorrhiza-like, and disease-antagonizing roles of entomopathogenic fungi.

## 1. Introduction

The hypocrealean fungi *Beauveria bassiana*, *Hirsutella thompsonii*, *Isaria fumosorosea*, *Metarhizium anisopliae*, *M. brunneum* and *M. robertsii* are known as entomopathogens of a wide range of arthropod pests. Several products of multiple hypocrealean fungi are commercially available as biopesticides for controlling pests in a variety of environments. When the fungal spores come in contact with their arthropod host, they germinate by producing a germ tube. An appressorium, an infective structure of the fungus, is formed at the end of the germ tube, exerting mechanical pressure and producing cuticle-degrading enzymes that help the fungus gain entry into the arthropod host. Once inside the host, the fungus multiplies, invades the host tissues, emerges from the host, and produces more spores to continue the infection cycle. While this entomopathogenic role of these fungi is well known, research in the past few years has expanded the understanding of fungi such as *B. bassiana* and its interaction with soil, plants, and other microbes in the environment [1]. This research has helped us understand how these fungi play an important role in promoting root development, plant growth, and health through a mycorrhiza-like and endophytic relationship with plants, inducing systemic resistance, and antagonizing plant pathogens. These non-pesticidal properties are critical in the fungal evolution, enabling them to survive in the soil and plant environments in the absence of an arthropod host. Based on recent studies and supporting data, *B. bassiana*, *Metarhizium* spp., and other hypocrealean fungi are considered as beneficial microbes that can improve soil structure and microbiome, probiotics that competitively displace harmful microorganisms, mycorrhiza-like fungi that improve nutrient and water absorption, and plant vaccines that induce systemic resistance. With a growing emphasis on sustainable food production systems, biologicals (including biocontrol agents, beneficial microbes, and biostimulants) have been gaining popularity in recent years because they reduce reliance on synthetic agricultural inputs, minimize environmental risks from pesticide residues or groundwater contamination with fertilizers, and improve water use efficiency. Apart from biopesticides, several commercial products based on beneficial bacteria, fungi, and yeasts are currently used as biostimulants; soil amendments; and soil, plant, or seed inoculants. Entomopathogenic fungi (EPF) appear to have many of these properties and can play a critical role in crop production on large and small farms as well as in home and community gardens. EPF promote plant growth, improve nutrient and water uptake, impart drought tolerance, stimulate plant defenses and induce resistance to biotic and abiotic stressors, adversely affect herbivores through nonpathogenic mechanisms, and antagonize plant pathogens through an endophytic and mycorrhiza-like relationship. This review emphasizes the non-entomopathogenic roles of EPF and how they provide additional benefits when applied to control arthropod pests. An increased understanding of these roles will help promote the use of EPF for sustainable food production.

## 2. Endophytic and Mycorrhiza-Like Interactions

In general, endophytic fungi and mycorrhizae have a symbiotic relationship with plants and usually have a negative impact on generalist herbivores [2]. While endophytes colonize the aerial plant parts, mycorrhizae (both ecto- and endomycorrhizae) form a relationship with the roots, acting as an extension of the root system. Both endophytes and mycorrhizae derive shelter and nutrition from the plants. Endophytism is similar to infection, without causing any disease. Endophytes offer protection against plant pathogens by inducing resistance [3,4] or increasing plant resistance to herbivores by stimulating the production of defensive compounds such as alkaloids [5,6]. Endophytic fungi can help plants withstand abiotic stressors [7], improve their interspecific and intraspecific competitive ability [8], and promote their growth [9]. Mycorrhizae help plants to withstand biotic and abiotic stressors such as pests, diseases, and drought, and they also help absorb nutrients [10,11,12]. Many of these interactions depend on the endophyte, mycorrhiza, plant, environment, herbivore, and plant pathogen in the phytobiome, and the communication among them [13,14]. Such symbiotic and mutualistic interactions are also seen with EPF, plants, herbivores, and plant pathogens.

## 3. Plant Defenses and Induced Resistance

Plants exhibit two kinds of induced resistance—systemic acquired resistance (SAR) and induced systemic resistance (ISR) as a defense towards biotic and abiotic stressors [15,16,17]. When plants are exposed to pathogens (virulent and avirulent), nonpathogenic microbes, or certain chemicals, SAR is triggered by the systemic accumulation of salicylic acid (a plant hormone) and pathogenesis-related proteins throughout the plant. Salicylic acid activates the SAR genes and prepares the plants for potential future infection by a variety of pathogens in a quick and effective manner. ISR, on the other hand, is triggered by nonpathogenic microbes that activate the jasmonic acid and ethylene pathways. Jasmonic acid production is also indicative of wounding in plants and can trigger pathogenesis-related proteins. The salicylate-induced pathway involves the production of antifungal pathogenesis-related proteins, such as chitinases, glucanases, and thaumatins, whereas the jasmonate-induced pathway involves the production of oxidative enzymes, such as peroxidases, polyphenol oxidases, and lipoxygenases. Nonpathogenic or plant growth-promoting bacteria that induce systemic resistance in plants include *Bacillus* spp., *Curtobacterium* sp., *Pseudomonas* spp., and *Serratia marcescens* (also an insect pathogen). Arbuscular mycorrhizal fungi such as *Glomus* spp. and *Piriformospora* spp. can also initiate mycorrhiza-induced resistance similar to SAR and ISR [18,19,20]. EPF appear to elicit such induced resistance and help plants withstand biotic and abiotic stressors [21,22]. Although several examples of EPF antagonizing plant pathogens or negatively impacting herbivores through nonpathogenic roles showed the effects without examining the physiological changes in the plant, some studies have provided additional insights into this mechanism of plant protection. Injecting the conidia of *B. bassiana* and *Lecanicillium dimorphum* and *L.* cf. *psalliotae* into date palm leaves induced the production of plant defense proteins [21]. EPF inoculation also influenced the regulation of proteins related to photosynthesis and energy metabolism. Dipping the roots of mouse-ear cress (also known as thale cress or *Arabidopsis*) in a *B. bassiana* conidial suspension influenced the expression of genes regulating pathogenesis, phytoalexin, jasmonic acid, and salicylic acid signaling pathways [22]. Although concentrations of jasmonic and salicylic acids did not increase, *B. bassiana* inoculation reduced the severity of disease caused by *Sclerotinia sclerotiorum*. Karthiba et al. [23] and Senthilraja et al. [24] demonstrated that *B. bassiana* along with *Pseudomonas fluorescens* increased the accumulation of pathogenesis-related proteins and other defense enzymes in rice and peanut, respectively. An accumulation of peroxidase and polyphenol oxidase in rice and phenylalanine ammonia-lyase, peroxidase, polyphenol oxidase, chitinases, β-1,3-glucanase, superoxide dismutase, catalase, lipoxygenase, and phenolics in peanuts increased as a result of these microbial treatments, protected the plants from insect pests and diseases, and improved plant growth and yields. Pineda et al. [25] presented several examples of endophytic fungi (*Acremonium* spp.), plant growth-promoting rhizobacteria (*Bacillus* spp., *Flavomonas* sp., *Pseudomonas* spp., and *S. marcescens*), and rhizobia (*Rhizobium* sp.) inducing resistance in several crops against a variety of pest insects. Pineda et al. [14] discussed several interactions of soil microbes, plants, and herbivores and explained how some recruit beneficial microbes for defense against herbivores by root exudation of sugars, organic acids, phytohormones, and secondary metabolites. Other plants growing in such soils can also show increased resistance to herbivore damage. These examples demonstrate that EPF and other beneficial microbes induce plant resistance to various stress factors.

## 4. Improved Nutrient Uptake and Plant Growth

*Beauveria bassiana* and other hypocrealean fungi are soilborne and have developed a relationship with plants. They are known to colonize several species of plants endophytically—living inside the plant. Examples of plants that were colonized by *B. bassiana* and other EPF include barley [26]; cassava [27]; cocoa [28]; forbs, grasses, green beans [29]; corn, soybean, tobacco, wheat [30,31]; strawberry [32]; and many other crop plants [33]. When fungi are applied through a soil drench, foliar application, or seed or transplant treatment, they colonize and benefit the plants in one or more ways. In return, EPF can survive in, on, or around the plants in the absence of their arthropod hosts and obtain nutrition from the plant.

When Behie et al. [34] demonstrated that *M. robertsii* translocated nitrogen from the larva of the waxmoth (*Galleria mellonella*) to the green bean, it developed the understanding of the insect–EPF–plant interaction in a different way. Behie and Bidochka [35] later showed that *B. bassiana*, *Metarhizium guizhouense*, *M. brunneum*, and *M. robertsii* translocated insect-derived nitrogen into green bean, soybean, switchgrass, and wheat and improved plant productivity. It appears that symbiotic soil fungi, and potentially EPF, upregulate nutrient transportation genes and related mechanisms in plants for limiting and nonlimiting soil nutrients [36]. In a later study, Behie et al. [37] demonstrated that plants provide photosynthate to the fungus in return of insect-derived nitrogen. They added ^13^CO_2_ to airtight plant growth chambers with green bean plants and later detected ^13^C in endophytic *M. robertsii* that also infected the *G. mellonella* larvae. 

Multiple studies showed improved nutrient uptake and plant growth promotion from endophytic *B. bassiana* and other EPF. Endophytic *B. bassiana* and *M. brunneum* improved iron availability, chlorophyll content, length of roots, and the abundance of fine roots in sorghum grown on calcareous substrate [38]. Soil application appeared to be a better inoculation method than seed treatment or foliar application in this study. In a potted plant study in California where cabbage was grown under simulated drought conditions, EPF, especially *B. bassiana*, improved plant growth, plant stand, shoot/root ratio, plant biomass, and nutrient absorption [39]. EPF, as they colonize the plant roots, act as root extensions and probably improve nutrient and water absorption and help plants withstand stress factors. A South African study showed that *B. bassiana* treatment did not impact greenhouse grapevine growth, but resulted in higher calcium and magnesium in leaf tissues [40]. Fungus-treated grapevines produced nine anti-insect compounds, compared to five anti-insect compounds in untreated vines. Application of *M. brunneum* encapsulated in polymer beads improved endophytism in potato by 13% when grown in nutrient-poor soil in a German study [41]. Improved nitrogen and phosphate content, plant biomass, leaf surface area, yields, and water use efficiency were observed from endophytic *M. brunneum*, although some parameters depended on the soil fertility.

In a preliminary study, a similar effect of *B. bassiana* was also seen on cotton seedlings where the water-holding capacity of the potting medium and the root length appeared to be positively impacted [42]. In a study conducted in Texas, seed treatment by *B. bassiana* and *Purpureocillium lilacinum* significantly enhanced dry plant biomass, development, and the number of squares in cotton [43]. In a study conducted in Denmark, corn seed treatment with *B. bassiana* promoted plant growth when nutrients were abundant, but not under low-nutrient conditions, further supporting its role in nutrient absorption [44]. Seed treatment of green beans with *B. bassiana* and *M. robertsii* alone and in combination improved some or all of the measured parameters of fresh and dry weights and lengths of roots and aerial parts in a Brazilian study [45]. In an Argentinian study, foliar application of *B. bassiana* in soybean [46] significantly improved: plant height; number of branches; weight of pods per branch and plant; number of pods per branch and plant; number of seeds per pods, branch, and plant; and seed weight and yield. Seed treatment of broad beans with *B. bassiana* and *M. brunneum* significantly increased the seedling emergence, plant height, number of leaf pairs, and fresh shoot and root weights in a study conducted in Jordan [47].

In a three-month raised bed study in Southern California, transplant plug treatment with *B. bassiana* improved plant health and growth, providing a better result than a commercial product with beneficial soil microbes [48]. In another study in a commercial strawberry field on the Central Coast, soil application of *B. bassiana*, *M. brunneum*, and *I. fumosorosea* products were compared with other beneficial microbial treatments for improving plant growth, health, and fruit yield [49]. Although a significant difference was not consistently seen, EPF appeared to have a positive impact on the measured parameters. Compared to the grower standard, *B. bassiana*, *M. brunneum*, and *I. fumosorosea* resulted in a 3.5%, 9.6%, and 8.3% increase in marketable fruit yield, respectively. In a more recent small plot study of strawberry, where EPF were compared with botanical and microbial fungicides in improving crop health, there was no impact on crop health; however, fruit yield from plots where *B. bassiana* and *I. fumosorosea* products were applied as foliar sprays was 14.4% and 4.4% higher than the untreated control, respectively (Dara, unpublished data). Isolates of *B. bassiana*, *I. fumosorosea*, and *Leacanicillium lecanii* endophytically colonized green beans through seed treatment and improved plant height and fresh biomass in a Chinese study [50]. One of the *B. bassiana* isolates appeared to be the best in promoting plant growth.

## 5. Impact on Herbivore Populations

When EPF are endophytic, they can indirectly affect herbivore populations through non-entomopathogenic mechanisms such as antibiosis and antixenosis and induced systemic resistance. EPF and their metabolites render the plants less favorable to herbivores and indirectly affect their fitness, longevity, and fecundity. This is different from the fungal infections caused through conidia or blastospores, since endophytic EPF grow as mycelia and do not produce infective structures within the plant tissues. Hence, endophytism is one of the additional roles of EPF. However, when EPF emerge from wounded plant tissues as a result of the feeding of chewing insects, conidiation and eventual infection may occur epiphytically. There are several examples of endophytic EPF negatively impacting herbivore infestations. Soil application of *B. bassiana* affected the green peach aphid (*Myzus persicae*) in potted strawberry plants [51]. Compared to the untreated control, plants where *B. bassiana* was applied through the soil had twice as many dead aphids. Dara and Dara [52] also found that strawberry plants with *M. brunneum* applied to the potting medium withstood twospotted spider mite (*Tetranychus urticae*) populations better than untreated plants. Endophytic colonization of *M. brunneum* could not be detected in these plants, and it was thought that either improved water absorption through a mycorrhiza-like effect helped plants resist pest pressure or the presence of fungus in the plant affected the mites. A significant reduction in *T. urticae* numbers was also seen on bean plants grown from seeds treated with *B. bassiana*, *M. robertsii*, and their combination, with no negative impact on predation by the predatory mite, *Phytoseiulus persimilis* [45]. Dash et al. [50] also reported the negative impact on *T. urticae* on bean plants grown from seeds treated with EPF. Compared to a 98% survival of *T. urticae* adult females on control bean plants, those treated with *B. bassiana*, *I. fumosorosea*, and *L. lecanii* had 68%, 72%, and 72% survival, respectively. Nymphal populations were significantly lower, and larval development, adult longevity, and female fecundity were also adversely affected on EPF-treated plants. 

In cotton, seed treatment with *B. bassiana* affected reproduction of the cotton aphid, *Aphis gossypii*, in the greenhouse and significantly reduced their populations in field studies [53]. Lopez and Sword [43] showed that *B. bassiana* seed treatment of cotton reduced the survival of larvae of the cotton bollworm, *Helicoverpa zea*, by 30%. In a German study, endophytic *B. bassiana* in grapevines reduced the size of the vine mealybug, *Planococcus ficus*, in laboratory assays and reduced larval numbers of the grape leafhopper, *Empoasca vitis* [54]. However, no positive impact on vine growth was reported. Similarly, Klieber and Reineke [55] did not see any impact of endophytic *B. bassiana* on tomato plant growth, but noticed a significant reduction on the fourth instar larvae of the tomato leafminer, *Tuta absoluta*. In a field study conducted in Kenya, seed treatment of green bean with *B. bassiana* reduced *Liriomyza* leafminer infestations and pupal numbers and improved yields without negatively affecting parasitoid populations [56]. A laboratory study in Slovakia evaluated the effect of endophytic *B. bassiana* strains in horse-chestnut saplings on the horse-chestnut leafminer, *Cameraria ohridella* [57]. In saplings with endophytic *B. bassiana*, leafminer damage was significantly less (area of damage was one-fifth of that of control saplings) and leafminer survival and size and weight of the pupae were significantly lower. A recent review of *B. bassiana* as an endophyte discussed various methods of inoculation, detection, and biocontrol potential, where most of the studies showed a negative or neutral effect on insect herbivores [58].

## 6. Disease Antagonism

Similar to having a negative impact on herbivores, EPF in the phyllosphere and rhizosphere or as endophytes can antagonize plant pathogens through parasitism, competition, and antibiosis or by inducing systemic resistance in the plant [59]. Antagonistic effect of *Beauveria* spp., in vitro or in vivo, against several plant pathogens, including *Botrytis cinerea*, *Fusarium oxysporum*, *Gaeumannomyces graminis*, *Pythium* sp., *Rhizoctonia solani*, and *Sptoria* sp., was reported in several earlier studies [60,61,62,63,64].

Protection against *Rhizoctonia solani* and *Pythium myriotylum* in cotton and tomato from seed treatment and against *Xanthomonas axonopodis* pv. *malvacearum* in cotton from soil application of *B. bassiana* was observed in multiple studies [65]. Increased plant growth, plant stand, or plant height and reduced disease severity were observed in *B. bassiana*-treated plants in the presence of plant pathogens in these studies. Competition for space, parasitism, or induced systemic resistance were thought to be the mechanisms of disease antagonism. Dara et al. [66] demonstrated that adding EPF to soil infested with *Fusarium oxysporum* f.sp. *vasinfectum* offered protection to cotton against the disease in a greenhouse study in California. Significant improvement in crop health was observed, especially with *B. bassiana*, which was even better than with botanical and microbial fungicides. In a preliminary potted plant study, Dara et al. [67] also found that California isolates of *B. bassiana* and *M. anisopliae* s.l. could improve strawberry plant health in the presence of *Macrophomina phaseolina*, a causal agent of the charcoal rot fungus. In a recent field study in California, alternating unformulated *B. bassiana* and *M. anisopliae* s.l. with chemical fungicides provided moderate control of *B. cinerea* and *Rhizopus* spp. in harvested strawberry [68]. In a study conducted in New Zealand, coating corn seed with *B. bassiana* and *Metarhizium* spp. provided protection against a disease as well as an insect pest [69]. There was a 22%–44% reduction in the root rot caused by *Fusarium graminearum*, along with up to 67% infection in the larvae of the scarabeid beetle *Costelytra giveni* (entomopathogenic effect) from the EPF treatments.

## 7. Conclusions

There are multiple levels of interactions of beneficial microbes, plants, phytopathogens, herbivores, and beneficial insects. There appears to be a complex network of communication among these organisms, and several examples discussed in this document explain how beneficial microbes help plants withstand biotic and abiotic stressors and how EPF have a similar relationship with plants. Many of the benefits of EPF are similar to those of the beneficial microbes used for improving soil structure and health, plant health, induced systemic resistance, drought or salt tolerance, and improved nutrient uptake. So far, this less-understood relationship of EPF with plants has great potential for exploring these organisms for non-entomopathogenic roles by improving plant growth, health, and yields. EPF can be good candidates for seed or transplant treatment and for soil application, especially where beneficial microbial populations are low. EPF can also be excellent pest management tools in home gardens and small urban farms where chemical pesticide use is less desirable. When used for pest management, EPF can also help with non-entomopathogenic roles and improve overall plant growth and health.
